# Short-Term Effects of Weight-Loss Meal Replacement Programs with Various Macronutrient Distributions on Gut Microbiome and Metabolic Parameters: A Pilot Study

**DOI:** 10.3390/nu15224744

**Published:** 2023-11-10

**Authors:** Seungmin Song, Jinyoung Shon, Woo-ri Yang, Han-Bit Kang, Keun-Ha Kim, Ju-Yeon Park, Sanghoo Lee, Sae Yun Baik, Kyoung-Ryul Lee, Yoon Jung Park

**Affiliations:** 1 Graduate Program in System Health Science and Engineering, Ewha Womans University, Seoul 03760, Republic of Korea; 2 Department of Nutritional Science and Food Management, Ewha Womans University, Seoul 03760, Republic of Korea; 3 Hyundai Greenfood Greating Laboratory, Yongin-si 16827, Republic of Korea; 4 SCL Healthcare Inc., Yongin-si 16954, Republic of Korea; 5 Hanaro Medical Foundation, Seoul 03159, Republic of Korea

**Keywords:** meal replacement, gut microbiome, short-term dietary intervention, calorie restriction, macronutrient distribution

## Abstract

It has emerged the gut microbiome is crucially linked to metabolic health and obesity. Macronutrient distribution has been discussed as a key parameter in weight-loss programs, but little is known about its impact on the gut microbiome. We investigated the effects of weight-loss meal replacement programs with different macronutrient ratios on the gut microbiota and metabolic parameters in subjects with overweight and obesity. Three low-calorie meal replacement programs with different ratios of carbohydrates, proteins, and lipids were designed: a balanced diet (Group B, 60:15:30), a high-lipid−low-carbohydrate diet (Group F, 35:20:55), and a protein-enriched diet (Group P, 40:25:35). Sixty overweight or obese participants were provided with the meals twice daily for 3 weeks. In all groups, diet intervention resulted in reduced body weight and BMI. The relative abundance of Bacteroidetes and Firmicutes phyla decreased and increased, respectively, which increased the *Firmicutes*/*Bacteroidetes* (F/B) ratio in all subjects, particularly in Groups B and P. Alpha- and beta-diversity were augmented at the phylum level in Group P. In conclusion, short-term interventions with weight-loss meal replacement programs increased butyrate-producing bacteria and the F/B ratio. Moreover, the protein-enriched diet significantly increased alpha- and beta-diversity compared to the balanced diet and the high-lipid−low-carbohydrate diet.

## 1. Introduction

About one-third of the world’s population is classified as overweight or obese, making it one of the biggest public health problems in the world [1]. Obesity is caused by excessive energy accumulation, and is one factor that increases the risk of chronic diseases, such as type 2 diabetes mellitus, cardiovascular disease, and cancer [2]. In the last two decades, functional compounds from plant and animal sources have been extensively explored to develop effective strategies for preventing these chronic diseases. However, there is a concern about the toxicity of high-dose functional foods during long-term and continuous consumption [3,4,5]. To mitigate this risk, meal-based diet intervention has emerged as a safe strategy for weight loss, and its optimal dietary pattern and macronutrient distribution have been explored in addition to calorie restriction. In the last few years, limited mobility due to coronavirus disease 2019 (COVID-19) has greatly affected eating habits, with dramatic increases in food delivery and the consumption of ready-made meals or home meal replacements [6,7]. Together with limited mobility, the changes in food choice have, at least in part, increased the risk of obesity and chronic disease. This situation has shed light on meal replacement programs based on healthy eating principles [8,9]. In our study, we provided weight-loss meal replacement programs with different macronutrient distributions to patients with obesity or overweight and investigated the effects on metabolic regulation and gut microbiome changes.

Macronutrient composition has long been debated in weight-loss dietary plans. Traditionally, dietary advice has focused on limiting fat intake to prevent obesity and chronic disease [10]. More recent research has shown that processed carbohydrates may also contribute to metabolic problems, leading to an increased interest in high-fat−low-carbohydrate diets, often accompanied by high protein, like the ketogenic diet [11]. In a systemic-review study, low-carbohydrate, high-protein, and Mediterranean diets were reported to be more effective in weight loss than low-fat and low-glycemic diets and moderate-fat diets with limited energy-maintained weight loss [12]. Although the debate is not conclusive on the optimal ratio of macronutrients, various strategies for metabolic health have been devised [13]. Intriguingly, multiple mouse studies on *ad libitum* feeding with various ratios of macronutrients showed that high-carbohydrate−low-protein diets yielded more positive results in metabolic regulation and longevity, despite an increase in body weight, compared to high-protein−low-carbohydrate diets [14]. The results indicate that further investigation is required to understand the effects of macronutrient distribution in diets on metabolic changes.

Evidence shows that the gut microbiome is a key factor influencing the risk of obesity and a useful biomarker for metabolic health [15,16,17]. Microbiome research is becoming the cornerstone of identifying individual variability in response to lifestyle or nutritional interventions for precision nutrition, with many studies being conducted on the effect of specific dietary factors on the diversity of the gut microbiome [18,19,20]. The most representative gut bacteria in humans are *Bacteroidetes*, *Firmicutes*, *Proteobacteria*, *Actinobacteria*, and *Verrucomicrobia* [17,21]. *Bacteroidetes* are gram-negative bacteria that ferment polysaccharides and indigestible carbohydrates, producing short-chain fatty acids (SCFAs). The SCFAs offer numerous beneficial effects in the gut [22,23]. Predominant genera within this group include *Bacteroides* and *Prevotella*. On the other hand, *Firmicutes* are a gram-positive bacteria characterized by their rigid or semi-rigid cell walls. They encompass over 200 different genera, including *Lactobacillus*, *Bacillus*, *Clostridium*, *Enterococcus*, and *Ruminicoccus*. *Firmicutes* are crucial in understanding the relationship between the gut microbiota and human health. Their enhanced ability to ferment and metabolize carbohydrates and lipids may link to the development of obesity [23,24]. Dietary compounds, such as fiber and fat, are one of the important modulators of gut microbiome composition [25]. Dietary fiber is a functional food component that can greatly impact the composition, diversity, and abundance of bacteria in the microbiome. Moreover, changes in the composition of the gut microbiome due to an increase in dietary fiber intake are concomitant with the relative expansion of specific bacterial populations [26].

Dysbiosis of the gut microbiome has been proven to be caused by diet-induced obesity, and clinical studies have shown that changes in the *Firmicutes*/*Bacteroidetes* (F/B) ratio are closely linked to obesity [27,28]. In several in vivo studies, changes in the abundance and physiological changes of the gut microbiome occurred during a high-fat diet. After 2-month-old male Sprague Dawley (SD) rats received a high-fat diet (60% fat) for 1 month, colonic macroscopic damage was significantly greater compared to the low-fat diet (10% fat) group, and the level of leptin was significantly increased [29]. In male C57BL/6NCrl mice fed with a high-fat diet (60% fat) for 12 weeks, changes in the diversity of dominant gut bacteria, bile acid and bilirubin metabolism, and amino acid and monosaccharide metabolism were observed [30]. Furthermore, in male SD rats that received a high-fat diet (60% fat) for 8 weeks, the F/B ratio increased, and metabolic functions, such as lipid and carbohydrate metabolism and glycan biosynthesis, differed from mice that received the normal diet (10% fat) [31]. The ketogenic diet, which is low in carbohydrates and high in fat, decreased *Bifidobacterium* due to an increase in ketone bodies, especially beta-hydroxybutyrate, which lowers inflammatory cell levels in intestinal fat and visceral fat, reducing insulin resistance and obesity [32]. In this context, a high-fat diet and a ketogenic diet not only change the phenotype, but also gut microbiome diversity and abundance, concomitant with physiologically metabolic changes. However, little is known about how diets with various macronutrient distributions affect gut microbiota. This study aimed to investigate the short-term effects of diet interventions with weight-loss meal replacement programs of various ratios of macronutrients on metabolic parameters and gut microbiota in subjects who are overweight or obesity.

## 2. Materials and Methods

### 2.1. Study Population

**Figure 1:**
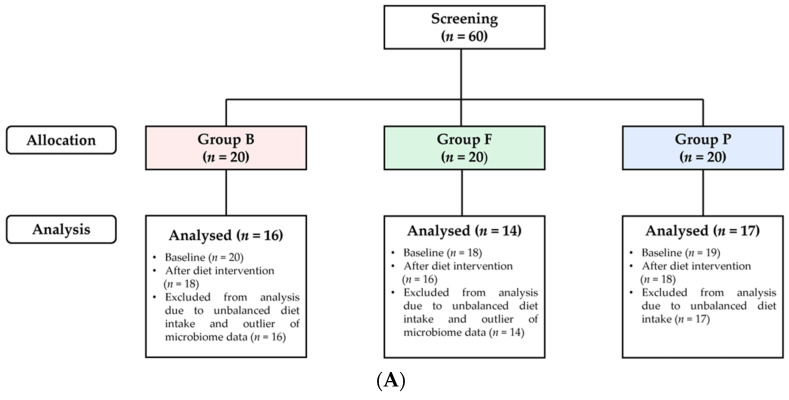

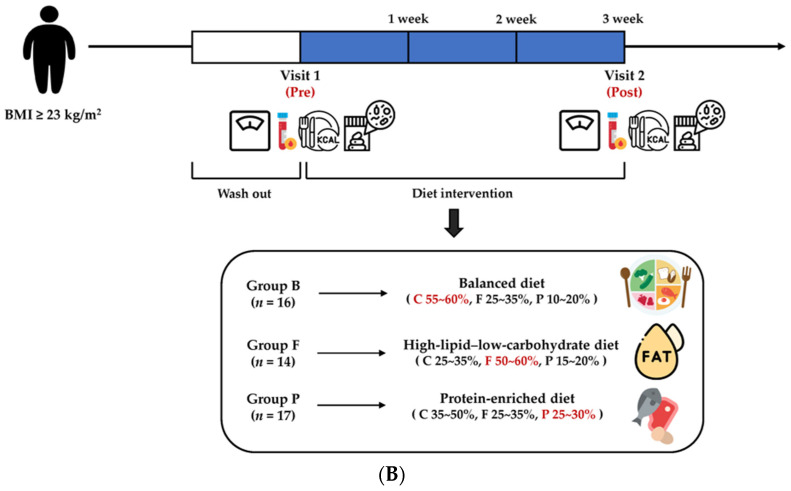
The flow of subjects’ selection and schematic representation of study design. (**A**) A flow diagram of subjects’ selection. There were 60 subjects in the weight-loss meal replacement program, and 47 subjects were finally analyzed. (**B**) Anthropometric measurements, dietary surveys, and blood and feces were sampled at visit 1 and visit 2 for subjects with BMI ≥ 23 kg/m^2^. The 3-week diet intervention was conducted with a balanced diet, a high-lipid–low-carbohydrate diet, and a protein-enriched diet.

This study recruited a total of 60 people by screening for customers at food service establishments operated by Hyundai Green Food in Seoul and Gyeonggi, Republic of Korea. As shown in Figure 1A, the subjects were assigned to three subgroups of 20 people each: Group B, Group F, and Group P. Subsequently, two participants in Group F and one in Group P were eliminated, and two in Group B and one in Group P were eliminated after the wash-out period (1 week) before diet intervention. After 3 weeks of diet intervention, 47 participants were finally analyzed by excluding subjects who had microbiome data outliers (i.e., microbiome data too low to read) or had consumed alcohol on 7 or more days during the intervention time. The exclusion criteria were as follows: (1) hypersensitive to certain foods or ingredients or have experienced severe food allergy reactions; (2) participated in other clinical studies 1 month prior to their first visit; (3) difficulty using smartphones; (4) judged by the researcher to be inappropriate to participate in this study; (5) difficulty taking appropriate samples during the study. All of the subjects agreed to participate in this study, signed a written consent form, and were adults aged between 20 and 65 years with a BMI ≥ 23 kg/m^2^. The elimination criteria were as follows: (1) if the diet is uncomfortable or unsatisfactory; (2) if there is an allergic reaction or other abnormal reaction after eating; (3) if the dietary intake rate is less than 50% and the dietary record is insufficient; (4) if sample collection is not possible; (5) if subjects find it impossible to participate in or continue the study. Participants were assigned using stratified randomization based on BMI. Body weight was recorded at the preliminary visit. This study was approved by the Ethics Committee of the Hanaro Medical Foundation (HMFIRB No. HNR2021-02, approval date 21 October 2021).

### 2.2. Diet Design

We constructed three meal plans with different ratios of calories from carbohydrate, lipid, and protein sources (Figure 1B). The balanced diet had a carbohydrate calorie ratio of ≥55% (Group B, *n* = 16), the high-lipid−low-carbohydrate diet had a lipid calorie ratio of ≥50% (Group F, *n* = 14), and the protein-enriched diet had a protein calorie ratio of ≥25% (Group P, *n* = 17). According to the 2020 Dietary Reference Intakes for Koreans, the daily energy intake of those aged 30~49 is 2200 kcal. The intervention diets provided in our study aimed at about 1100 kcal for two out of three meals, and the other meal was freely consumed. The balanced diet consisted of 4~5 kinds of side dishes, the high-lipid−low-carbohydrate diet was served in the form of salad, and the protein-enriched diet was served in the form of a one-dish meal, and it was composed differently for each meal, so it did not overlap for a week. Experimental diets were eaten for a total of 3 weeks (21 days), and two meals (lunch and dinner) with snacks were provided per day. Participants were asked to eat at regular times every day, and snacks were eaten freely during the day. As shown in Table 1, to minimize the difference according to calories in all three diets, the calories per meal were adjusted to 530 kcal to provide 1060 kcal ± 5% per day.

### 2.3. Clinical Assessments

As shown in Figure 1B, body measurements (weight, height, waist circumference, and BMI) were determined using an InBody 720 (Biospace Co., Seoul, Republic of Korea) at the preliminary visit (−1 week), visit 1 (0 week), and visit 2 (3 weeks). The diet survey was conducted using the Recommended Food Score (RFS) and Meats, Eggs, Dairy, Fried foods, fat in baked goods, Convenience foods, fats added at the Table, and Snacks (MEDFICTS) during visit 1. Sleep duration and exercise were investigated at visit 1 and 2. Meal and lifestyle education was assessed at the preliminary visit and visit 1, and a food diary was prepared for 3 days before visits 1 and 2. In the food diary, wake-up/bedtime, exercise, drinking, and smoking were also recorded. For biochemical markers, blood was collected after 12 h of fasting during visits 1 and 2 and processed for serum and plasma.

### 2.4. Microbiome Analysis

Subjects were asked to take a stool sample at visits 1 and 2, and it was stored at −80 °C. Fusion primers targeting the V3–V4 region of the bacterial 16S rRNA gene were selected for PCR amplification, using 341 forward and 805 reverse primers. Low-quality sequences were filtered using the DADA2 pipeline in the QIIME 2 package version 2021.4 (https://qiime2.org, accessed on 20 September 2022), and the amplicon sequence variants were generated. Taxonomy analysis was performed to confirm the relative frequency of the subjects’ gut microbiome at the phylum, family, and genus levels, and analysis of the alpha- and beta-diversity were performed using QIIME 2.

### 2.5. Statistical Analysis

We performed one-way ANOVA to confirm that there were no significant differences in lifestyle, anthropometric, and biochemical indicators, and diet intake among the three groups at pre- and post-diet intervention. A paired *t*-test was performed to confirm significant differences in lifestyle indicators, anthropometric indicators, biochemical indicators, dietary intake, and gut microbiome data at the phylum level and in the F/B ratio pre- and post-diet intervention, and all were analyzed using SAS 9.4 (SAS Institute, Cary, NC, USA). A violin plot representing alpha-diversity, PCoA plot representing beta-diversity, and heatmap representing Spearman’s rank order correlation analysis were constructed using R software version 4.2.2 (R Foundation for Statistical Computing, Vienna, Austria). Significant differences in alpha-diversity at pre- and post-diet intervention in each group were analyzed with the Kruskal−Wallis test using the R program, and beta-diversity at pre-and post-diet intervention in the total subjects and each group were analyzed using permutational multivariate analysis (PERMANOVA) using QIIME 2.

## 3. Results

### 3.1. Characteristics of the Subjects

A total of 47 subjects (45 men, 2 women) participated in this study, including 16 in Group B, 14 in Group F, and 17 in Group P. Lifestyle, anthropometric, and biochemical indicators were investigated before diet intervention with the weight-loss meal replacement programs (Table 2). The mean age of the total subjects was 36.0 ± 4.3, physical activity was 124.6 ± 86.5 kcal/day, and sleep duration was 6.60 ± 0.73 h/day. From the dietary history of the total subjects, the mean RFS score was 19.9 ± 8.7, and the mean MEDFICTS score was 66.5 ± 11.8. The mean body weight of the total subjects was 81.9 ± 12.2 kg, with a mean BMI of 26.7 ± 3.0 kg/m^2^, and a mean waist-to-hip ratio (WHR) of 0.9 ± 0.1. The mean values for biochemical markers related to liver function of the total subjects were gamma-glutamyl transferase (γ-GTP) 29.6 ± 19.5 IU/L, total bilirubin 0.7 ± 0.3 mg/dL, aspartate aminotransferase (AST) 26.9 ± 13.6 IU/L, alanine aminotransferase (ALT) 30.4 ± 20.2 IU/L, lactate dehydrogenase (LDH) 189.6 ± 30.4 IU/L, and alkaline phosphatase (ALP) 63.6 ± 13.2 U/L. Further analyses of the total subjects showed the mean level of creatinine, associated with kidney function, was 0.9 ± 0.1 mg/dL, and the lipid profile showed mean levels of 129.2 ± 63.1 mg/dL for triglycerides (TG), 203.3 ± 29.0 mg/dL for total cholesterol (T-Cho), 51.9 ± 9.7 mg/dL for high-density lipoprotein (HDL), and 139.4 ± 26.8 mg/dL for low-density lipoprotein (LDL). When comparing the values of biochemical markers with normal values, there were no clinical problems and no indicators with significant differences among the three groups.

### 3.2. Weight-Loss Meal Replacement Program Effects on Anthropometric and Biochemical Indicators

There were no significant differences in the diet intake and percentage of calories among the three groups before diet intervention. As shown in Table 3, the daily calorie intake after diet intervention in the total subjects was significantly decreased −569.1 ± 431.1 kcal/day, carbohydrate −62.5 ± 55.9 g/day, protein −18.4 ± 20.9 g/day, and fat −14.8 ± 23.7 g/day. In Group B, the percentage of calories from carbohydrates increased by 11.2 ± 7.2%, and the percentage of calories from protein and fat decreased. The daily intake of fat in Group F increased by 11.0 ± 13.4 g/day, and the percentage of calories increased by 15.5 ± 5.8%. In Group P, the daily intake of calories, carbohydrate, and fat decreased significantly, but the protein remained the same, and the percentage of calories increased significantly, by 5.2 ± 3.1%.

After the 3-week weight-loss meal replacement program, changes in lifestyle, anthropometric, and biochemical indicators were investigated (Table 4). Among the anthropometric factors, body weight, BMI, fat-free mass (FFM), and relative body weight (RBW) significantly decreased in the total subjects and each of the three groups, and WHR increased, albeit not significantly. Among the biochemical markers related to liver function, the levels of ALP and γ-GTP after diet intervention were significantly lowered in the total subjects and in each of the three groups. After diet intervention, the ALT level was significantly lower in the total subjects (−4.8 ± 17.2 IU/L), and the LDH level was lower in the total subjects (−17.1 ± 31.8 IU/L) and Group F (−27.9 ± 39.1 IU/L). However, total bilirubin was significantly higher in the total subjects (0.1 ± 0.3 mg/dL) and Group B (0.2 ± 0.3 mg/dL) after diet intervention, and the level of liver function markers in the total subjects was lowered except for total bilirubin, indicating that the weight-loss meal replacement program improved liver function. Among the lipid profile markers, the levels of TG and T-Cho were significantly lower in the total subjects, especially T-Cho in Group P (−11.1 ± 15.9 mg/dL). Conversely, the HDL levels decreased, especially in Group B (−4.4 ± 6.9 mg/dL) and Group P (−4.4 ± 5.0 mg/dL), and the LDL levels increased. This is consistent with another study in which the HDL level temporarily decreased during a short-term, very-low-calorie diet (VLCD) but returned after adaptation to the intervention diet [33].

In addition, after diet intervention, the total protein level was significantly lowered in the total subjects (−0.2 ± 0.3 g/dL) and in Group F (−0.2 ± 0.2 g/dL) and Group P (−0.2 ± 0.3 g/dL), and high-sensitivity C-reactive protein (hsCRP) was significantly decreased in the total subjects (−0.7 ± 1.3 mg/L) and Group F (−0.7 ± 1.0 mg/L). Leptin and adiponectin were lowered in the total subjects. In particualr, leptin was significantly lowered in Group F (−3.9 ± 5.7 ng/mL) and Group P (−2.0 ± 2.2 ng/mL), and fibroblast growth factor 21 (FGF21) was significantly lowered in the total subjects (−39.0 ± 79.1 pg/mL) and Group F (−56.3 ± 86.7 pg/mL).

### 3.3. Changes in Gut Microbiome by Diet Intervention

**Figure 2:**
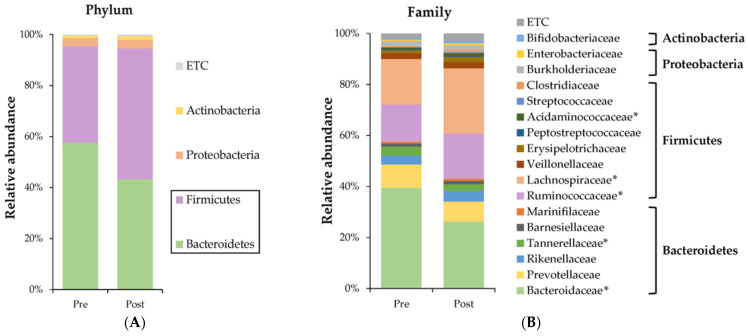

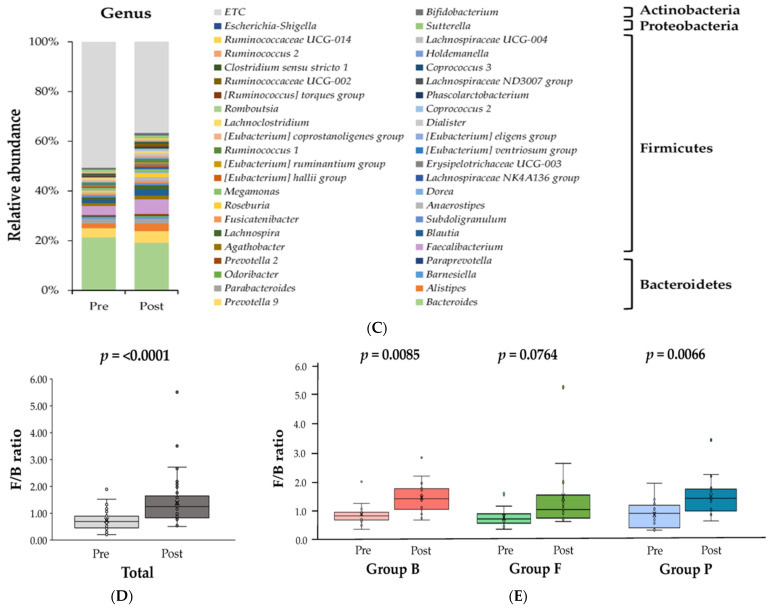
Changes in gut microbiome by diet intervention in Group B, F, and P. The relative abundance of (**A**) phylum level, (**B**) family level, (**C**) genus level, and (**D**) *Firmicutes*-to-*Bacteroidetes* (F/B) ratio at pre- and post-intervention in total subjects, and (**E**) F/B ratio at pre- and post-intervention by Group B, F, and P. In (**A**), *Firmicutes* and *Bacteroidetes* were used to obtain the F/B ratio of (**D**,**E**). Classified microbiome levels with relative abundances above a cutoff level of 0.2% are indicated. Gray bars represent microbiome with a relative abundance of <0.2%. Data presented as mean ± standard error. Total (*n* = 47), Group B (*n* = 16), Group F (*n* = 14), and Group P (*n* = 17). Statistical significance was measured using a paired *t*-test (*p* < 0.05). Asterisk (*) indicated a microbiome showing a significant difference.

To investigate the effects of diet intervention on the subjects, the fecal microbiota was characterized by 16S rRNA gene sequencing. The compositions of the gut microbiome at the phylum, family, and genus levels and the F/B ratio are presented in Figure 2A–D and Table 5. The main bacterial phyla were *Bacteroidetes* and *Firmicutes*, followed by *Proteobacteria* and *Actinobacteria*. In the total subjects (Figure 2A), the relative abundance of *Bacteroidetes* decreased and *Firmicutes* increased. Post diet intervention, the significant changes at the family level (Figure 2B) were the decrease in *Bacteroidaceae* and the increases in *Ruminococcaceae* and *Lachnospiraceae*. At the genus level (Figure 2C), a significant decrease in *Bacteroides* and increases in *Faecalibacterium* and *Blautia* were most noteworthy. As shown in Figure 2D,E and Table 5, *Bacteroidetes* decreased and *Firmicutes* increased after diet intervention in all three groups, resulting in augmented F/B ratios. The F/B ratio significantly increased by 0.7 ± 1.0% in the total subjects, 0.6 ± 0.8% in Group B, and 0.7 ± 0.9% in Group P. *Proteobacteria* showed no significant difference between pre- and post-diet intervention, whereas *Actinobacteria* increased significantly in the total subjects (0.5 ± 1.2%) after diet intervention.

**Figure 3:**
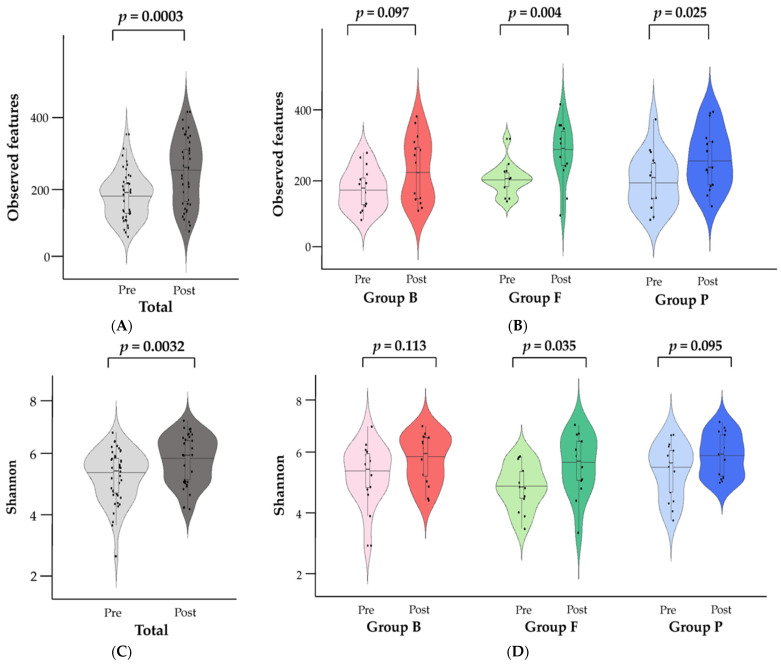

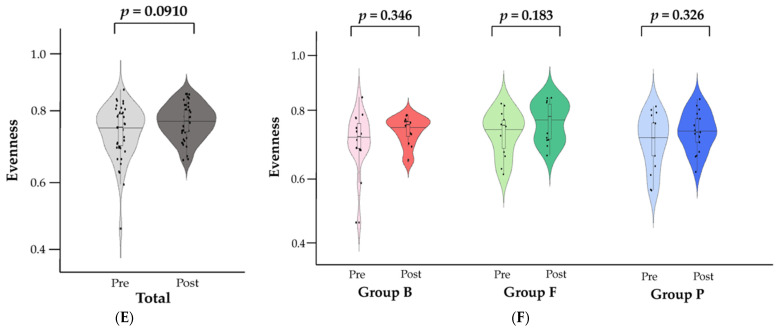
Changes in gut bacterial alpha-diversity at the phylum level by diet intervention in Group B, F, and P. Alpha-diversity violin plots to visualize the difference in microbiota at the phylum level between pre- and post-diet intervention. Alpha-diversity comparisons of (**A**,**B**) observed features, (**C**,**D**) Shannon index, and (**E**,**F**) species evenness in the fecal microbiota among Group B, F, and P (Kruskal−Wallis test). There was no significant difference in the change (post−pre) values among the three groups.

To investigate the alpha-diversity in each group pre and post diet intervention, observed features and the Shannon index were used to evaluate the species richness of the gut microbiome, and the species evenness was also analyzed (Figure 3). In the total subjects (Figure 3A,C,E), species richness increased significantly post diet intervention compared to pre diet intervention (observed features *p* = 0.003, Shannon index *p* = 0.0032), but there was no significant difference in species evenness (*p* = 0.0910). When comparing the alpha-diversity pre- and post-diet intervention by group, there was no significant difference in all groups in species evenness (Figure 3F). In the species richness analysis, there was a significant difference in the observed features in Group F (*p* = 0.004) and Group P (*p* = 0.025) and a significant difference in the Shannon index only in Group F (*p* = 0.035). Therefore, there was no difference in the evenness of the gut microbiome after the weight-loss meal replacement program, but a significant difference in microbiome diversity had occurred in both Group F and Group P.

**Figure 4:**
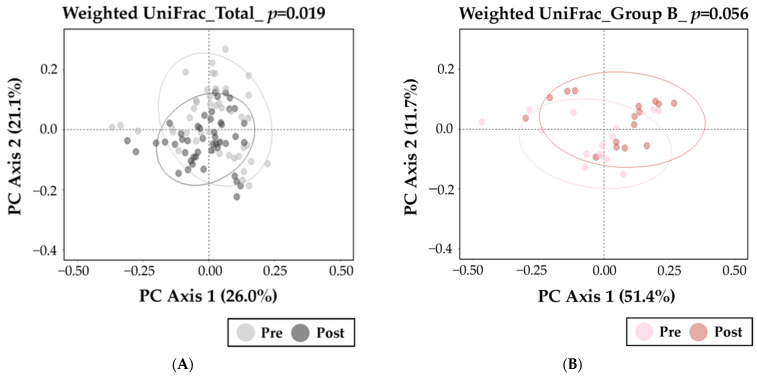

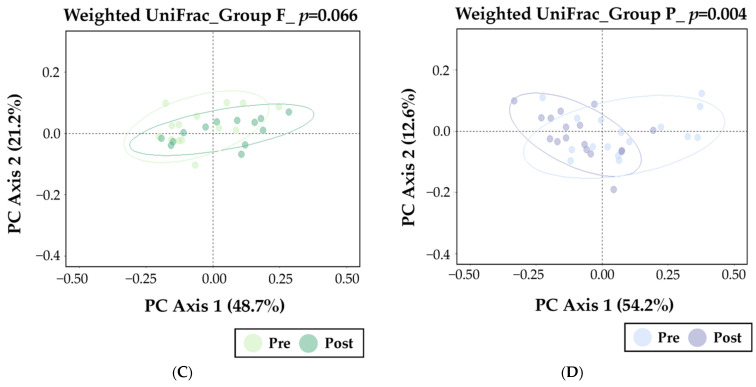
Changes in gut bacterial beta-diversity at the phylum level by diet intervention in Group B, F, and P. PCoA plots of beta-diversity derived using weighted UniFrac to visualize the difference in microbiota at the phylum level between pre- and post-diet intervention for total subjects (**A**), Group B (**B**), Group F (**C**), and Group P (**D**). The statistical significance of weighted UniFrac was measured using permutational analysis of variance (PERMANOVA).

Beta-diversity was measured using weighted UniFrac to assess differences in microbiome composition pre and post diet intervention of the total subjects, and significant differences were analyzed by PERMANOVA (Figure 4). There was a significant difference in the microbiome composition pre and post diet intervention in the total subjects (Figure 4A, *p* = 0.019). Figure 4B–D shows beta-diversity pre and post diet intervention in each group. Only Group P (Figure 4D, *p* = 0.004) showed a significant difference in the pre- to post-intervention change in the composition of the gut microbiome, not Group B (*p* = 0.056, Figure 4B) or Group F (*p* = 0.066, Figure 4C).

### 3.4. Correlation between Microbiome and Lifestyle, Anthropometric, Biochemical, and Diet Markers

**Figure 5:**
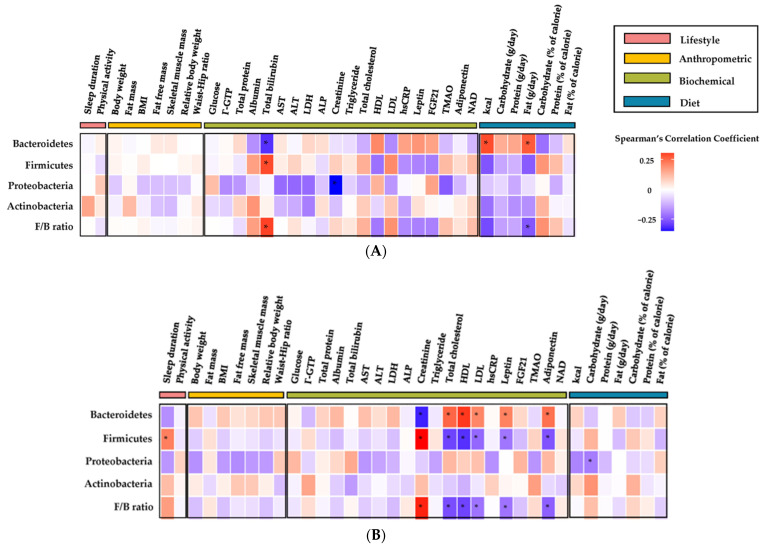
Spearman’s rank order correlation analysis of lifestyle, anthropometric, biochemical, diet, and microbiome. Spearman’s rank order correlation matrix of a total of 41 significant observations, including lifestyle (red), anthropometric (yellow), biochemical (green), diet (blue), and microbiome data among Group B, F, and P. (**A**) Spearman’s rank order correlation determined between lifestyle, anthropometric, biochemical, diet, and microbiome indicators to assess subjects’ pre- and post-diet intervention status. (**B**) Spearman’s rank order correlation of diet intervention-induced changes (post−pre) in lifestyle, anthropometric, biochemical, diet, and microbiome indicators to assess the subjects’ change status. BMI, body mass index; γ-GTP, gamma-glutamyl transferase; AST, aspartate aminotransferase; ALT, alanine aminotransferase; LDH, lactate dehydrogenase; ALP, alkaline phosphatase; HDL, high-density lipoprotein; LDL, low-density lipoprotein; hsCRP, high-sensitivity C-reactive protein; FGF21, fibroblast growth factor 21; TMAO, trimethylamine-*N*-oxide; NAD; nicotinamide adenine dinucleotide. Asterisk (*) means variables with a correlation of 0.25 ≥ or ≤−0.25.

Spearman’s rank order correlation was computed to assess the correlation of the gut microbiome with lifestyle, anthropometric, biochemical, and diet markers (Figure 5). In Figure 5A, total bilirubin showed a negative correlation with *Bacteroidetes* and a positive correlation with *Firmicutes*. Accordingly, the F/B ratio also showed a positive correlation. In addition, *Proteobacteria* showed a strong negative correlation with creatinine. Among the diet markers, calories (kcal) and fat (g/day) showed positive correlations with *Bacteroidetes*, and fat (g/day) showed a significant negative correlation with the F/B ratio. Figure 5B reveals the changes in other markers related to the gut microbiome. Creatinine showed a negative correlation with *Bacteroidetes* and a positive correlation with *Firmicutes*, while the lipid profile (T-Cho, HDL, and LDL), leptin, and adiponectin showed positive correlations with *Bacteroidetes* and negative correlations with *Firmicutes*. Accordingly, the F/B ratio showed a correlation in the same direction as *Firmicutes*. A significant result in the change of the diet markers was the negative correlation between carbohydrate (g/day) and *Proteobacteria*.

**Figure 6:**
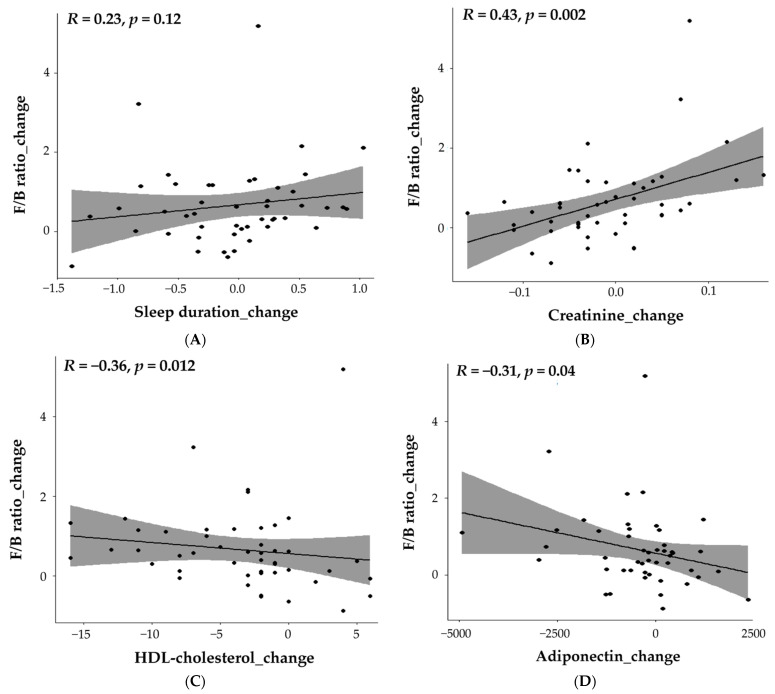
Scatter plots and Spearman’s rank correlation coefficient between change in F/B ratio and change in sleep duration, creatinine, HDL-cholesterol, and adiponectin. To investigate Spearman’s rank correlation coefficient between the change in F/B ratio and change in sleep duration, creatinine, HDL-cholesterol, and adiponectin in the total subjects, it was indicated by a scatter plot. Scatter plot of (**A**) sleep duration, (**B**) creatinine, (**C**) HDL-cholesterol, and (**D**) adiponectin, the parameters that were most closely related to the F/B ratio according to Figure 5B.

In the total subjects, the correlation between the change in the F/B ratio and the change in sleep duration, one of the lifestyle markers, was significant (r = 0.23, Figure 6A). Likewise, the correlation between the change in the F/B ratio and the change in the biochemical indicators was significant for creatinine (r = 0.43), HDL-cholesterol (r = −0.36), and adiponectin (r = −0.31) in the total subjects (Figure 6B–D).

## 4. Discussion

In this study, we characterized anthropometric and biochemical parameters, diet intake, and microbiome data changes mediated by different weight-loss meal replacement programs with different proportions of macronutrients (carbohydrate, fat, and protein) in subjects who were overweight or obesity. Among the results for changes in the gut microbiome, changes in the F/B ratio, known as an obesity marker, were observed. While an increase in the F/B ratio is known to be related to weight gain [24,34], clinical studies have stated that it is difficult to determine health status from the F/B ratio, and a correlation of the F/B ratio with obesity has not been identified [35,36]. In this study, the F/B ratio increased in all groups, which was coincident with similar studies [35,36,37,38], even though body weight was lost due to calorie-restricted diets. *Bacteroidetes* decreased by 14.3% at the phylum level (*p* < 0.0001) due to a 13.3% decrease in *Bacteroidaceae* at the family level (*p* < 0.0001) and a 13.2% decrease in *Bacteroides* at the genus level (*p* = 0.0002). *Firmicutes* increased by 13.6% at the phylum level (*p* < 0.0001) due to a 3.1% increase in *Ruminococcaceae* at the family level (*p* = 0.0389), a 7.7% increase in *Lachnospiraceae* (*p* < 0.0001) at the family level, and a 3.3% increase in *Faecalibacterium* at the genus level (*p* = 0.0079). *Ruminococcaceae* and *Lachnospiraceae* are butyrate-producing bacteria belonging to the *Firmicutes* phylum. Butyrate, produced by most of the complex carbohydrates and plant polysaccharides consumed by humans, is one of the SCFAs, which are important metabolites for maintaining intestinal homeostasis.

Butyrate-producing bacteria can promote a beneficial metabolism by fermenting dietary fibers in the intestinal lumen [39,40,41]. Dietary fiber shapes the gut microbiota composition, and fermentable dietary fiber increases the intestinal production of SCFA, which may help prevent obesity [42,43]. In our study, fiber intake was significantly increased in the total subjects (4.3 ± 7.0, *p* = 0.0001, Table S1) and Group B (10.9 ± 5.3, *p* < 0.0001, Table S1) after diet intervention. In the mouse model, when a refined high-fat diet (rHFD) was ingested, body weight and fat mass increased significantly compared to mice that ingested chow and a refined low-fat diet (rLFD), but there was no significant difference in F/B ratio between rLFD and rHFD. The reason is the different fiber content in these two diets [37]. Furthermore, research has shown a higher F/B ratio, lower lipid level, and less liver fat accumulation in mice that ingested a purified-fiber diet compared to a purified-starch diet [38]. Our results highlight the increased F/B ratio, accompanied by the positive health outcomes. This may be attributed to a notable rise in butyrate-producing Firmicutes within the gut microbiota, stemming from a higher fiber intake in the calorie-restricted intervention diets. Another potential explanation relates to energy adaptation. *Firmicutes* are known to be more adept than *Bacteroidetes* at extracting energy from food [42,44]. Given the short duration of our intervention, our findings may depict the microbiome’s adaptive phase, becoming more efficient in energy utilization under calorie-restricted diets. This notion is reinforced by the consistent changes in the F/B ratio across the three groups, all of which followed energy-restricted meal plans.

Alpha- and beta-diversity analyses at the phylum level were performed to compare the diversity of intestinal microorganisms within or between groups. Microbial diversity in the intestine is associated with health status, and when diversity is reduced, disease conditions, such as inflammatory bowel disease, may occur [45]. A low-diversity microbiome is suggestive of dysbiosis and has been linked to being overweight or obesity [46,47,48]. Our study confirmed that weight and BMI decreased in subjects who were overweight or obesity during a calorie-restricted diet. Moreover, alpha-diversity (observed features, Shannon index) increased in the total subjects and Group F and Group P. Compared to the balanced diet, which had a uniform proportion of macronutrients, the high-lipid–low-carbohydrate diet, which had a 50~60% increase in fat content, and the protein-enriched diet, which had a 25~30%, increase in protein content, are thought to have increased species richness (alpha-diversity) because these diets included fruits, salads, and nuts. In particular, fruits, salads, and nuts were provided daily in the high-lipid–low-carbohydrate diet and more frequently than in the protein-enriched diet.

A diet rich in fruits and vegetables has been suggested to be associated with changes in intestinal microbial composition and is known to play an important role in improving intrinsic ecology [49,50]. Higher intakes of fruit have been associated with greater gut microbiome diversity and a lower risk of type 2 diabetes and are supported by dietary recommendations for increasing fruit consumption to prevent this disease [49]. In addition, the Mediterranean diet, a plant-based diet that emphasizes fruits, vegetables, and nuts, is favorably associated with an increased abundance of *Ruminococcaceae* and *Lachnospiraceae* families [51], which can be associated with an increase in the F/B ratio after intervention with the Mediterranean diet. Alpha-diversity (Shannon index) was significantly increased by a calorie-restricted high-protein (30% protein) diet group compared to a calorie-restricted normal protein (15% protein) diet, indicating that a high-protein diet can modulate the gut microbiome in obesity [52]. In our study, it was found that the species richness increased in the groups that consumed fruits and vegetables (Group F and P), suggesting that this was an important difference compared to Group B, and alpha-diversity increased significantly (observed features and Shannon index), especially in subjects that consumed a lot of fruits and vegetables (Group F). Conversely, only Group P induced a significant difference in the pre- and post-diet intervention change in beta-diversity. Therefore, the protein-enriched diet seems to affect alpha- and beta-diversity more than the others. While the macronutrient distribution did have an impact on microbial diversity, the significant shifts we noted in the microbiome seemed to be more linked to the weight loss diet than to the specific diet composition. Even though the three meal plans differed in the magnitude of changes observed, key alterations, like those in the F/B ratio, were consistently seen across all groups following the intervention.

The correlation of the microbiome markers (*Bacteroidetes*, *Firmicutes*, *Proteobacteria*, and *Actinobacteria*) with the F/B ratio and lifestyle, anthropometric, biochemical, and diet markers was analyzed using Spearman’s rank order. As shown in Figure 5A, total bilirubin and creatinine, as biochemical markers, and intake of calories (kcal) and fat (g/day), as diet markers, were highly related to the status of the static microbiome. Total bilirubin showed a negative correlation with *Bacteroidetes* and positive correlations with *Firmicutes* and the F/B ratio. Bilirubin, a component of the heme catabolism pathway essential for liver function, is conjugated and secreted into the bile by the liver and catabolized by microbes in the intestine to urobilinoids before excretion. There are four bacteria known to reduce bilirubin to urobilinoids: *Clostridium ramosum*, *Clostridium perfringens*, *Clostridium difficile*, and *Bacteroides fragilis*, which are essential for normal gut function or can play an important role in the development of intestinal disease [53]. Of the four bacteria, *Clostridium spp.* belong to the phylum *Firmicutes* [54,55,56] and *Bacteroides spp.* belong to *Bacteroidetes* [57]. In our study, although the above bacteria were not included in the top 15 at the genus level, the relative abundance of *Firmicutes* increased and *Bacteroidetes* decreased at the phylum level, supporting the role of the intestinal microbiota in bile acid metabolism. In addition, looking at the markers related to liver function in Table 4, the levels in the total subjects and each group decreased after diet intervention except for total bilirubin, which increased in the total subjects, Group B, and Group F. Similarly, serum bilirubin levels increased by 18~45% when people with overweight or obesity lost weight after a short-term, calorie-restricted diet [58]. It has been reported that an increase in serum bilirubin levels in people who are overweight or obesity can protect other organs and directly affect fat tissue and fat secretion patterns, thereby improving various obesity-mediated or obesity-related diseases [58,59].

In Figure 5B, when looking at markers highly associated with microbiome shifts induced by diet intervention, most were biochemical markers of lipid profiles, especially T-Cho, HDL-cholesterol, and LDL-cholesterol. In addition, creatinine, leptin, adiponectin, sleep duration (a lifestyle marker), and carbohydrate (g/day; a diet marker) showed significant correlations. It is not known how serum creatinine with the highest correlation relates to microbial bacteria, which should be further studied. In addition, the lipid profile showed a high correlation, which can be related to a short diet intervention. Table 4 shows that the HDL levels decreased after a calorie-restricted diet in all groups. Although calorie-restricted diets are generally expected to increase HDL levels, other studies have shown that VLCDs reduce weight and, as a temporary phenomenon, HDL levels also decrease before returning to pre-VLCD levels or improving [33]. Similar to our study, a 28-day diet intervention of 800 kcal/day showed significant weight loss, but the level of HDL was 1.05 ± 0.04 mmol/L before diet intervention and decreased to 0.09 ± 0.03 mmol/L after the intervention [60].

This pilot study has proposed a potential benefit of the effects of macronutrient intake patterns on the gut microbiome based on a short-term meal-based intervention. However, several points should be considered when designing future research on the optimal macronutrient distributions for health maintenance. While our weight-loss meal replacement programs reduced body weight or BMI in people who were overweight or obesity during short-term diet intervention, further study is needed to evaluate whether that effect is temporary or permanent. In addition, weight-loss effects and changes in HDL-cholesterol levels should be checked even after long-term intervention. Another limitation is that the sample size was less than 20 in each of the three groups, which was insufficient to be representative of the population who are overweight or obesity. A small sample size can reveal clinically significant results, but because other statistically significant results may be insufficient [61], it is necessary to increase the sample size in further studies to determine the effective ratio of macronutrient intake. There was also no information on the micronutrients in our designed weight-loss meals. In this regard, when diet intervention is performed in vivo, the same raw materials are used for the diets, but in a clinical study, the dietary food items used to achieve the macronutrient distribution ratio are different. As the macronutrient distribution ratio is changed, the food composition changes, especially in micronutrients, but this was not considered in our study. Additionally, as shown in Table S2, according to the actual intake of micronutrients consumed by the subjects, some nutrients did not reach the recommended nutrient intake. Future studies on meal plans should consider micronutrients in study design and analysis.

## 5. Conclusions

Our study demonstrated that a moderate calorie-restricted meal intervention with various macronutrient intake patterns significantly reduced body weight, BMI, and the levels of TG and T-Cho, and improved hepatic metabolic parameters, except total bilirubin. The changes in the parameters were not significantly different between the macronutrient patterns. The intervention increased the intestinal distribution ratio of butyrate-producing bacteria among the most abundant *Firmicutes* in the body and elevated the F/B ratio, regardless of different macronutrient ratios. Furthermore, it increased the microbiome diversity, in particular, after intervention with the protein-enriched diet, compared to the high-lipid–low-carbohydrate diet and that with the balanced diet. The findings suggested that microbiome alterations may be more sensitive to dietary macronutrient patterns than other anthropometric and biochemical measures. Future studies should focus on long-term interventions.

## Figures and Tables

**Table 1:** Composition of the three intervention diets.

	Energy (kcal/Day)	Carbohydrate (g/Day)	Protein (g/Day)	Fat (g/Day)
Group B	1044	155 (59.3%)	39 (14.9%)	33 (28.5%)
Group F	1117	93 (33.3%)	49 (17.5%)	66 (53.6%)
Group P	1038	107 (41.2%)	67 (25.7%)	40 (34.5%)

Group B, balanced diet; Group F, high-lipid–low-carbohydrate diet; Group P, protein-enriched diet. Each value in parentheses is energy from carbohydrate, fat, and protein.

**Table 2:** Baseline characteristics of the three groups of subjects in the dietary intervention study.

	Total (*n* = 47)	Diet Intervention			*p*-Value
		Group B (*n* = 16)	Group F (*n* = 14)	Group P (*n* = 17)	
**Age (years)**	36.0 ± 4.3	36.4 ± 5.2	35.6 ± 3.9	35.8 ± 3.8	0.8626
**Male, *n* (%)**	45 (95.7)	15 (93.8)	13 (92.9)	17 (100)	
**Physical activity (kcal/day)**	124.6 ± 86.5	144.1 ± 73.8	120.3 ± 91.8	109.8 ± 94.6	0.5201
**Sleep duration (hours)**	6.60 ± 0.73	6.65 ± 0.93	6.54 ± 0.60	6.60 ± 0.64	0.9108
**Dietary history**					
RFS *	19.9 ± 8.7	19.0 ± 9.8	18.1 ± 7.9	22.2 ± 8.2	0.3912
MEDFICTS **	66.5 ± 11.8	62.3 ± 10.3	65.9 ± 13.1	71.1 ± 10.9	0.0951
**Anthropometric parameters**					
Height (cm)	174.9 ± 5.9	174.1 ± 5.8	175.4 ± 6.6	175.2 ± 5.9	0.8157
BW (kg)	81.9 ± 12.2	79.9 ± 10.4	84.6 ± 15.4	81.5 ± 11.2	0.5821
BMI (kg/m^2^)	26.7 ± 3.0	26.3 ± 2.3	27.3 ± 3.5	26.6 ± 3.3	0.6461
FM (%)	25.3 ± 5.9	25.9 ± 6.2	26.2 ± 5.0	24.0 ± 6.4	0.5505
FFM (%)	60.8 ± 7.2	59.0 ± 6.4	62.1 ± 9.2	61.5 ± 6.1	0.4515
SMM (kg)	34.4 ± 4.3	33.3 ± 3.9	35.2 ± 5.5	34.8 ± 3.7	0.4475
RBW (%)	21.3 ± 13.3	19.7 ± 10.0	24.0 ± 14.6	20.7 ± 15.2	0.6662
WHR	0.9 ± 0.1	0.9 ± 0.1	0.9 ± 0.1	0.9 ± 0.1	0.4716
**Biochemical parameters**					
**Liver function**					
γ-GTP (IU/L)	29.6 ± 19.5	28.9 ± 22.8	29.9 ± 17.0	29.9 ± 19.2	0.9877
Total Bilirubin (mg/dL)	0.7 ± 0.3	0.7 ± 0.2	0.6 ± 0.3	0.8 ± 0.4	0.2370
AST (IU/L)	26.9 ± 13.6	22.8 ± 10.7	33.6 ± 18.3	25.2 ± 9.8	0.0727
ALT (IU/L)	30.4 ± 20.2	27.7 ± 24.2	35.3 ± 19.0	28.8 ± 17.4	0.5570
LDH (IU/L)	189.6 ± 30.4	185.8 ± 31.2	195.6 ± 35.6	188.1 ± 26.0	0.6659
ALP (U/L)	63.6 ± 13.2	64.1 ± 12.7	63.4 ± 16.0	63.3 ± 12.1	0.9816
**Kidney function**					
Creatinine (mg/dL)	0.9 ± 0.1	0.9 ± 0.1	0.9 ± 0.2	0.9 ± 0.1	0.9449
**Lipid profiles**					
TG (mg/dL)	129.2 ± 63.1	120.4 ± 65.2	129.3 ± 58.1	137.4 ± 67.6	0.7505
T-Cho (mg/dL)	203.3 ± 29.0	202.6 ± 30.4	200.0 ± 32.3	206.7 ± 26.0	0.8144
HDL (mg/dL)	51.9 ± 9.7	56.2 ± 9.3	48.1 ± 9.2	50.9 ± 9.3	0.0599
LDL (mg/dL)	139.4 ± 26.8	135.5 ± 27.8	139.1 ± 29.8	143.2 ± 24.2	0.7176
**Others**					
Glucose (mg/dL)	92.3 ± 9.3	90.3 ± 7.0	94.6 ± 12.9	92.2 ± 7.7	0.4651
Total protein (g/dL)	7.4 ± 0.4	7.3 ± 0.4	7.4 ± 0.4	7.4 ± 0.3	0.4694
Albumin (g/dL)	4.8 ± 0.2	4.8 ± 0.2	4.7 ± 0.1	4.8 ± 0.2	0.0809
hsCRP (mg/L)	1.4 ± 1.6	1.9 ± 2.1	1.7 ± 1.6	0.7 ± 0.5	0.0819
Leptin (ng/mL)	12.1 ± 11.8	14.6 ± 16.7	13.4 ± 10.8	8.8 ± 4.8	0.3313
Adiponectin (ng/mL)	6919.7 ± 4066.9	6699.6 ± 3485.8	6761.7 ± 4218.0	7256.7 ± 4638.1	0.9153
FGF21 (pg/mL)	140.6 ± 96.2	136.3 ± 70.8	154.1 ± 120.9	133.5 ± 98.9	0.8240
TMAO (umol/L)	4.5 ± 5.5	5.5 ± 7.9	5.1 ± 5.0	2.9 ± 2.3	0.3550
NAD (ug/mL)	21.8 ± 3.0	22.2 ± 3.3	21.5 ± 3.6	21.8 ± 2.1	0.7839

All values are expressed as mean ± standard deviation. Significant differences in baseline values among Group B, F, and P were compared using one-way ANOVA. BW, body weight; BMI, body mass index; FM, fat mass; FFM, fat-free mass; SMM, skeletal muscle mass; RBW, relative body weight; WHR, waist-to-hip ratio; γ-GTP, gamma-glutamyl transferase; AST, aspartate aminotransferase; ALT, alanine aminotransferase; LDH, lactate dehydrogenase; ALP, alkaline phosphatase; HDL, high-density lipoprotein; LDL, low-density lipoprotein; hsCRP, high-sensitivity C-reactive protein; FGF21, fibroblast growth factor 21; TMAO, trimethylamine-N-oxide; NAD, nicotinamide adenine dinucleotide. * Recommended Food Score (RFS): the scores range from 0 to 47, and a higher score indicates a better-quality diet. ** Meats, Eggs, Dairy, Fried foods, fat in baked goods, Convenience foods, fats added at the Table, and Snacks (MEDFICTS): step 2 diet (score < 40, <7% of energy from saturated fat and <200 mg cholesterol), step 1 diet (score 40–69, 8–10% of energy from saturated fat and <300 mg cholesterol), high-fat diet (score > 70).

**Table 3:** Diet intake of subjects and diet intervention change data of subjects.

	Total (*n* = 47)	*p*-Value	Diet Intervention						*p*-Value (ANOVA)
			Group B (*n* = 16)	*p*-Value	Group F (*n* = 14)	*p*-Value	Group P (*n* = 17)	*p*-Value	
Energy (kcal/day)	−569.1 ± 376.6	**<0.0001**	−735.2 ± 441.0	**<0.0001**	−452.0 ± 343.6	**0.0003**	−509.3 ± 293.7	**<0.0001**	0.6353
Carbohydrate (g/day)	−62.5 ± 55.9	**<0.0001**	−51.0 ± 64.6	**0.0065**	−89.5 ± 47.9	**<0.0001**	−51.2 ± 48.1	**0.0005**	0.1739
Protein (g/day)	−18.4 ± 20.9	**<0.0001**	−36.1 ± 17.5	**<0.0001**	−17.1 ± 17.1	**0.0025**	−2.7 ± 12.6	0.3860	0.9641
Fat (g/day)	−14.8 ± 23.7	**<0.0001**	−31.0 ± 20.3	**<0.0001**	11.0 ± 13.4	**0.0089**	−20.8 ± 14.3	**<0.0001**	0.7501
Carbohydrate (% of calorie)	1.0 ± 11.2	0.5397	11.2 ± 7.2	**<0.0001**	−10.3 ± 8.1	**0.0004**	0.7 ± 6.6	0.6601	0.1401
Protein (% of calorie)	1.4 ± 3.8	**0.0127**	−1.8 ± 1.9	**0.0018**	0.5 ± 1.4	0.2063	5.2 ± 3.1	**<0.0001**	0.3230
Fat (% of calorie)	3.2 ± 9.4	**0.0233**	−3.1 ± 4.3	**0.0103**	15.5 ± 5.8	**<0.0001**	−0.9 ± 4.2	0.3774	0.1210

All values are expressed as mean ± standard deviation. The *p*-values comparing the specific timeline values at pre- and post-diet intervention in the total subjects, Group B, Group F, and Group P were measured using paired *t*-test. The *p*-values measured using ANOVA showed a significant difference in the pre-value among the three groups.

**Table 4:** Lifestyle, anthropometric, and biochemical change data of subjects.

	Total (*n* = 47)	*p*-Value	Diet Intervention					
			Group B (*n* = 16)	*p*-Value	Group F (*n* = 14)	*p*-Value	Group P (*n* = 17)	*p*-Value
**Lifestyle parameters**								
Physical activity (kcal/day)	−0.9 ± 73.7	0.9341	1.5 ± 54.0	0.9144	−0.9 ± 1.8	0.4639	12.4 ± 67.6	0.4600
Sleep duration (hours)	−0.1 ± 0.5	0.5345	−0.1 ± 0.7	0.5576	0.1 ± 0.4	0.2435	−0.1 ± 0.5	0.2534
**Anthropometric parameters**								
BW (kg)	−1.4 ± 1.3	**<0.0001**	−1.2 ± 0.9	**<0.0001**	−1.8 ± 1.6	**0.0012**	−1.1 ± 1.2	**0.0013**
BMI (kg/m^2^)	−0.5 ± 0.4	**<0.0001**	−0.4 ± 0.3	**<0.0001**	−0.6 ± 0.5	**0.0008**	−0.4 ± 0.4	**0.0016**
FM (%)	−0.3 ± 1.0	0.0616	−0.6 ± 1.1	0.0557	−0.02 ± 0.8	0.9198	−0.2 ± 1.0	0.4220
FFM (%)	−0.8 ± 1.0	**<0.0001**	−0.5 ± 0.8	**0.0335**	−1.3 ± 1.3	**0.0021**	−0.7 ± 0.8	**0.0015**
SMM (kg)	−0.4 ± 0.6	**<0.0001**	−0.3 ± 0.5	0.0827	−0.8 ± 0.8	**0.0028**	−0.4 ± 0.5	**0.0089**
RBW (%)	−2.1 ± 1.9	**<0.0001**	−1.9 ± 1.4	**0.0001**	−2.7 ± 2.3	**0.0008**	−1.7 ± 1.9	**0.0017**
WHR	0.003 ± 0.01	0.1295	0.001 ± 0.01	0.6091	0.003 ± 0.01	0.3019	0.004 ± 0.02	0.3109
**Biochemical parameters**								
**Liver function**								
γ-GTP (IU/L)	−8.8 ± 10.0	**<0.0001**	−8.7 ± 9.8	**0.0028**	−10.4 ± 11.4	**0.0044**	−7.5 ± 9.6	**0.0054**
Total Bilirubin (mg/dL)	0.1 ± 0.3	**0.0413**	0.2 ± 0.3	**0.0216**	0.1 ± 0.2	0.1134	−0.01 ± 0.3	0.9060
AST (IU/L)	−2.6 ± 18.2	0.3424	−1.8 ± 5.2	0.1840	−7.2 ± 19.9	0.1975	0.6 ± 24.1	0.9211
ALT (IU/L)	−4.8 ± 12.8	**0.0138**	−4.3 ± 10.7	0.1342	−7.5 ± 14.3	0.0718	−3.0 ± 13.6	0.3751
LDH (IU/L)	−17.1 ± 31.8	**0.0006**	−11.1 ± 25.2	0.0983	−27.9 ± 39.1	**0.0191**	−13.8 ± 30.3	0.0784
ALP (U/L)	−6.8 ± 5.6	**<0.0001**	−5.3 ± 5.0	**0.0007**	−9.9 ± 5.1	**<0.0001**	−5.8 ± 5.8	**0.0008**
**Kidney function**								
Creatinine (mg/dL)	−0.01 ± 0.1	0.3813	−0.02 ± 0.1	0.3661	−0.01 ± 0.1	0.7034	−0.003 ± 0.1	0.8614
Lipid profiles								
TG (mg/dL)	−17.1 ± 40.09	**0.0054**	−13.9 ± 28.4	0.0696	−17.4 ± 48.8	0.2044	−19.8 ± 43.7	0.0808
T-Cho (mg/dL)	−4.8 ± 17.2	0.0642	−1.6 ± 17.8	0.7206	−0.6 ± 17.0	0.8895	−11.1 ± 15.9	**0.0109**
HDL (mg/dL)	−3.6 ± 5.3	**<0.0001**	−4.4 ± 6.9	**0.0218**	−1.6 ± 2.7	0.0491	−4.4 ± 5.0	**0.0025**
LDL (mg/dL)	1.5 ± 16.3	0.5332	5.1 ± 16.2	0.2296	3.1 ± 16.7	0.4948	−3.2 ± 15.8	0.4100
**Others**								
Glucose (mg/dL)	−0.3 ± 7.4	0.7977	−1.5 ± 4.3	0.1792	−0.5 ± 11.0	0.8678	1.1 ± 6.0	0.4807
Total protein (g/dL)	−0.2 ± 0.3	**0.0001**	−0.1 ± 0.3	0.2565	−0.2 ± 0.2	**0.0043**	−0.2 ± 0.3	**0.0078**
Albumin (g/dL)	0.01 ± 0.1	0.6596	0.03 ± 0.1	0.4506	0.0 ± 0.1	1.0000	0.0 ± 0.2	1.0000
hsCRP (mg/L)	−0.7 ± 1.3	**0.0008**	−1.2 ± 1.9	0.0267	−0.7 ± 1.0	**0.0148**	−0.2 ± 0.4	0.0733
Leptin (ng/mL)	−3.2 ± 4.8	**<0.0001**	−3.7 ± 5.8	0.0215	−3.9 ± 5.7	**0.0226**	−2.0 ± 2.2	**0.0019**
Adiponectin (ng/mL)	−416.2 ± 1287.8	**0.0317**	−271.6 ± 791.9	0.1903	−227.0 ± 1450.2	0.5682	−708.3 ± 1525.9	0.0737
FGF21 (pg/mL)	−39.0 ± 79.1	**0.0015**	−31.7 ± 64.4	0.0681	−56.3 ± 86.7	**0.0304**	−31.8 ± 87.4	0.1531
TMAO (umol/L)	−0.4 ± 6.3	0.6497	−0.9 ± 9.8	0.7295	−1.6 ± 3.3	0.0919	1.0 ± 3.6	0.2846
NAD (ug/mL)	−0.2 ± 2.4	0.6471	0.9 ± 3.0	0.2355	−0.9 ± 1.8	0.0838	−0.6 ± 1.9	0.2256

All values are expressed as mean ± standard deviation. Significant differences between pre- and post-values in Group B, F, and P were compared using paired *t*-test. BW, body weight; BMI, body mass index; FM, fat mass; FFM, fat-free mass; SMM, skeletal muscle mass; RBW, relative body weight; WHR, waist-to-hip ratio; γ-GTP, gamma-glutamyl transferase; AST, aspartate aminotransferase; ALT, alanine aminotransferase; LDH, lactate dehydrogenase; ALP, alkaline phosphatase; TG, triglyceride; T-Cho, total cholesterol; HDL, high-density lipoprotein; LDL, low-density lipoprotein; hsCRP, high-sensitivity C-reactive protein; FGF21, fibroblast growth factor 21; TMAO, trimethylamine-N-oxide; NAD, nicotinamide adenine dinucleotide.

**Table 5:** The percentage of major phyla in the gut microbiome of subjects.

	Total (*n* = 47)	*p*-Value	Diet Intervention					
			Group B (*n* = 16)	*p*-Value	Group F (*n* = 14)	*p*-Value	Group P (*n* = 17)	*p*-Value
***Bacteroidetes* (%)**	−14.3 ± 17.2	**<0.0001**	−13.5 ± 15.4	**0.0032**	−13.6 ± 20.3	**0.0260**	−15.6 ± 16.9	**0.0016**
***Firmicutes* (%)**	13.6 ± 16.5	**<0.0001**	12.5 ± 16.2	**0.0073**	12.3 ± 16.5	**0.0151**	15.6 ± 17.6	**0.0022**
***Proteobacteria* (%)**	0.2 ± 4.5	0.7045	0.4 ± 4.5	0.7178	0.8 ± 6.4	0.6325	−0.4 ± 2.2	0.4698
***Actinobacteria* (%)**	0.5 ± 1.2	**0.0087**	0.5 ± 1.4	0.1380	0.5 ± 0.9	0.0647	0.4 ± 1.3	0.1965
**F/B ratio (%)**	0.7 ± 1.0	**<0.0001**	0.6 ± 0.8	**0.0085**	0.7 ± 1.5	0.0764	0.7 ± 0.9	**0.0066**

All values are expressed as mean ± standard deviation. The *p*-values comparing specific timeline values at pre- and post-diet intervention in total subjects, Group B, Group F, and Group P were measured using a paired *t*-test. F/B ratio; *Firmicutes*/*Bacteroidetes* ratio.

## Data Availability

Data are contained within the article.

## Supplementary Materials

The following supporting information can be downloaded at: https://www.mdpi.com/article/10.3390/nu15224744/s1, Table S1: Micronutrient change data of subjects.; Table S2: Micronutrient intakes of subjects in the diet intervention change data of subjects.
