# Supplementary material for: Short-Term Effects of Weight-Loss Meal Replacement Programs with Various Macronutrient Distributions on Gut Microbiome and Metabolic Parameters: A Pilot Study
Source: Nutrients. 2023 Nov 10;15(22):4744. doi: 10.3390/nu15224744 (PMC10675061; doi:10.3390/nu15224744)
Supplement: Supplementary file 1 [file nutrients-15-04744-s001.zip › nutrients-2675152-supplementary-done.pdf]

nutrients

Table S1. Micronutrient change data of subjects.

	Total (*n* = 47)	*p*-Value	Diet Intervention					
			Group B (*n* = 16)	*p*-Value	Group F (*n* = 14)	*p*-Value	Group P (*n* = 17)	*p*-Value
**Sodium (mg)**	−1508.8 ± 777.0	**<0.0001**	−1624.9 ± 768.4	**<0.0001**	−1709.0 ± 856.2	**<0.0001**	−1234.6 ± 676.6	**<0.0001**
**Potassium (mg)**	246.5 ± 442.7	**0.0004**	64.3 ± 451.4	0.5774	487.6 ± 463.1	**0.0017**	219.5 ± 336.7	**0.0162**
**Phosphorus (mg)**	−77.1 ± 179.9	**0.0051**	−160.7 ± 195.9	**0.0051**	17.5 ± 205.0	0.7550	−76.4 ± 90.0	**0.003**
**Iodine (ug)**	−4.6 ± 31.7	0.3263	−12.6 ± 47.2	0.3008	2.3 ± 18.4	0.6448	−2.7 ± 20.5	0.5943
**Calcium (mg)**	−53.6 ± 130.0	**0.0051**	−109.7 ± 114.8	**0.0017**	24.2 ±161.3	0.5837	−69.9 ± 76.1	**0.0016**
**Vitamin A (RE)**	42.6 ± 47.1	**<0.0001**	18.0 ± 52.7	0.1911	60.5 ± 47.0	**0.0003**	50.9 ± 32.1	**<0.0001**
**Retinol (ug)**	−4.4 ± 74.1	0.6883	−52.6 ± 62.5	**0.0042**	49.4 ± 80.2	**0.0382**	−3.3 ± 44.9	0.7685
**β-carotene (ug)**	434.8 ± 796.8	**0.0005**	246.3 ± 838.7	0.2585	495.9 ± 1025.8	0.0937	561.8 ± 504.7	**0.0003**
**Vitamin D (ug)**	−1.5 ± 2.4	**<0.0001**	−2.0 ± 1.6	**0.0002**	−1.0 ± 2.6	0.1703	−1.5 ± 2.9	**0.0495**
**Vitamin E (ug)**	4.0 ± 2.9	**<0.0001**	4.2 ± 2.2	**<0.0001**	3.4 ± 4.2	**0.0098**	4.4 ± 2.3	**<0.0001**
**Vitamin K (ug)**	108.9 ± 47.2	**<0.0001**	94.3 ± 19.3	**<0.0001**	155.2 ± 57.4	**<0.0001**	84.4 ± 25.5	**<0.0001**
**Vitamin B1 (mg)**	0.4 ± 0.4	**<0.0001**	0.2 ± 0.3	**0.0142**	0.7 ± 0.4	**<0.0001**	0.4 ± 0.4	**0.0035**
**Vitamin B2 (mg)**	−0.1 ± 0.3	**0.0251**	−0.1 ± 0.3	0.2853	0.0 ± 0.3	0.9760	−0.2 ± 0.2	**0.0049**
**Niacin (mg)**	0.4 ± 3.8	0.4263	−2.1 ± 4.3	0.0703	2.8 ± 3.0	**0.0040**	0.9 ± 2.4	0.1265
**Pantothenic acid (mg)**	0.9 ± 0.6	**<0.0001**	0.9 ± 0.5	**<0.0001**	1.0 ± 0.6	**<0.0001**	0.9 ± 0.7	**<0.0001**
**Vitamin B6 (mg)**	0.2 ± 0.1	**<0.0001**	0.2 ± 0.1	**0.0007**	0.2 ± 0.1	**<0.0001**	0.2 ± 0.1	**<0.0001**
**Biotin (ug)**	8.8 ± 3.5	**<0.0001**	9.2 ± 2.3	**<0.0001**	8.6 ± 5.3	**<0.0001**	8.6 ± 2.7	**<0.0001**
**Folate (ug)**	86.4 ± 45.1	**<0.0001**	67.5 ± 32.1	**<0.0001**	103.6 ± 60.2	**<0.0001**	90.0 ± 36.1	**<0.0001**
**Vitamin B12 (ug)**	−0.8 ± 2.6	**0.044**	−1.2 ± 3.0	0.1274	0.4 ± 1.4	0.2387	−1.3 ± 2.6	**0.0484**
**Vitamin C (mg)**	24.3 ± 33.7	**<0.0001**	17.4 ± 42.3	0.1209	26.6 ± 41.9	**0.0337**	29.0 ± 9.5	**<0.0001**
**TS (g)**	−3.0 ± 15.7	0.1988	−5.6 ± 12.6	0.0981	−1.9 ± 17.6	0.6966	−1.5 ± 17.3	0.7273
**TAA (mg)**	−458.3 ± 8571.4	0.7156	−2572.9 ± 9186.7	0.2802	488.0 ± 9055.0	0.8433	752.4 ± 7659.5	0.6908
**EAA (mg)**	−357.3 ± 4097.1	0.5529	−1526.7 ± 4175.0	0.1642	296.8 ± 4366.4	0.8032	204.7 ± 3788.4	0.8265
**NEAA (mg)**	−92.8 ± 4287.6	0.8827	−1284.9 ± 4420.9	0.2632	362.6 ± 4623.7	0.7738	654.2 ± 3868.3	0.4956
**TFA (g)**	3.7 ± 12.0	**0.0411**	−0.7 ± 10.5	0.7992	13.5 ± 9.8	**0.0002**	−0.2 ± 10.8	0.9278
**EFA (g)**	2.2 ± 2.4	**<0.0001**	2.3 ± 1.8	**0.0001**	2.0 ± 3.4	**0.0451**	2.2 ± 2.0	**0.0004**
**SFA (g)**	−8.4 ± 7.2	**<0.0001**	−11.7 ± 6.4	**<0.0001**	−1.6 ± 5.8	0.3123	−10.8 ± 5.4	**<0.0001**
**MUFA (g)**	4.7 ± 6.0	**<0.0001**	1.5 ± 5.6	0.3031	10.3 ± 4.1	**<0.0001**	3.2 ± 4.6	**0.0125**
**PUFA (g)**	1.8 ± 2.4	**<0.0001**	1.9 ± 1.8	**0.0008**	1.8 ± 3.4	0.0639	1.7 ± 2.0	**0.0031**
**Water (g)**	−135.5 ± 188.1	**<0.0001**	−92.4 ± 188.2	0.0684	−178.2 ± 226.4	**0.0114**	−141.0 ± 152.9	**0.0016**
**Ash (g)**	−2.4 ± 3.0	**<0.0001**	−2.6 ± 3.2	**0.0057**	−1.9 ± 3.6	0.0671	−2.5 ± 2.3	**0.0004**
**Fiber (g)**	4.3 ± 7.0	**0.0001**	10.9 ± 5.3	**<0.0001**	1.9 ± 6.1	0.2755	0.1 ± 4.2	0.8935
**Cholesterol (mg)**	−85.0 ± 82.0	**<0.0001**	−132.6 ± 62.2	**<0.0001**	−35.4 ± 86.8	0.1512	−81.0 ± 71.7	**0.0003**
**Chlorine (mg)**	72.3 ± 67.2	**<0.0001**	49.5 ± 29.4	**<0.0001**	74.9 ± 109.2	**0.0235**	91.7 ± 39.5	**<0.0001**
**Magnesium (mg)**	24.6 ± 38.4	**<0.0001**	3.3 ± 37.6	0.7311	35.8 ± 44.3	**0.0098**	35.6 ± 25.0	**<0.0001**
**Fluorine (ug)**	5.0 ± 3.5	**<0.0001**	2.2 ± 1.9	**0.0004**	5.3 ± 2.1	**<0.0001**	7.3 ± 3.8	**<0.0001**
**Iron (mg)**	−9.5 ± 7.0	**<0.0001**	−9.0 ± 6.6	**<0.0001**	−8.0 ± 7.9	**0.0023**	−11.1 ± 6.7	**<0.0001**
**Zinc (mg)**	−0.4 ± 2.1	0.2046	−1.0 ± 2.5	0.1123	0.7 ± 1.9	0.1620	−0.7 ± 1.6	0.0822
**Copper (mg)**	0.2 ± 0.1	**<0.0001**	0.2 ± 0.1	**0.0007**	0.4 ± 0.1	**<0.0001**	0.2 ± 0.1	**<0.0001**
**Manganese (mg)**	0.6 ± 0.4	**<0.0001**	0.8 ± 0.4	**<0.0001**	0.4 ± 0.5	**0.0063**	0.5 ± 0.3	**<0.0001**
**Selenium (ug)**	−3.8 ± 15.6	0.1009	−9.2 ± 18.6	0.0651	2.6 ± 11.3	0.4014	−4.0 ± 14.3	0.2687
**Molybdenum (ug)**	5.4 ± 16.2	**0.026**	−2.8 ± 20.2	0.5897	4.6 ± 15.0	0.3032	14.1 ± 6.2	**<0.0001**

All values are expressed as mean ± SD. Baseline values among Group B, F, and P were compared by paired *t*-test. TS, total sugars; TAA, total amino acids; EAA, essential amino acids; NEAA, non-essential amino acids; TFA, total fatty acid; EFA, essential fatty acid; SFA, saturated fatty acid; MUFA, mono-unsaturated fatty acid; PUFA, poly-unsaturated fatty acid.

Table S2. Micronutrient intakes of subjects in the diet intervention before and after.

	Total (*n* = 47)			Diet Intervention									*p*-Value (ANOVA)
				Group B (*n* = 16)			Group F (*n* = 14)			Group P (*n* = 17)			
	Pre	Post	*p*-Value	Pre	Post	*p*-Value	Pre	Post	*p*-Value	Pre	Post	*p*-Value	
**Sodium (mg)**	3823.4 ± 902.7	2314.6 ± 445.1	**<0.0001**	3997.1 ± 934.2	2372.1 ± 500.2	**<0.0001**	3894.1 ± 986.6	2185.1 ± 461.4	**<0.0001**	3601.7 ± 804.4	2367.0 ± 375.7	**<0.0001**	0.4359
**Potassium (mg)**	1741.9 ± 554.3	1988.4 ± 344.1	**0.0004**	1834.0 ± 699.9	1898.3 ± 399.0	0.5774	1740.8 ± 592.0	2228.4 ± 314.9	**0.0017**	1656.0 ± 350.8	1875.5 ± 196.1	**0.0162**	0.6634
**Phosphorus (mg)**	863.2 ± 225.4	786.1 ± 122.7	**0.0051**	913.2 ± 250.9	752.6 ± 139.1	**0.0051**	807.0 ± 285.7	824.5 ± 136.3	**0.7550**	862.5 ± 124.1	786.1 ± 87.5	**0.0030**	0.4454
**Iodine (ug)**	34.6 ± 38.0	30.0 ± 16.6	0.3263	36.5 ± 53.0	23.9 ± 11.6	0.3008	30.6 ± 22.1	32.9 ± 21.7	**0.6448**	36.1 ± 33.1	33.4 ± 14.9	0.5943	0.8977
**Calcium (mg)**	414.1 ± 157.8	358.7 ± 109.8	0.0051	417.4 ± 156.3	307.7 ± 96.4	**0.0017**	437.0 ± 200.7	461.2 ± 80.1	**0.5837**	392.2 ± 122.5	322.2 ± 86.8	**0.0016**	0.7381
**Vitamin A (RE)**	43.0 ± 45.1	85.6 ± 15.9	**<0.0001**	54.2 ± 50.2	72.2 ± 6.3	0.1911	43.6 ± 52.4	104.1 ± 13.9	0.0003	32.0 ± 31.5	82.8 ± 6.6	**<0.0001**	0.3739
**Retinol (ug)**	124.5 ± 77.4	120.1 ± 66.7	0.6883	133.3 ± 66.4	80.7 ± 60.3	**0.0042**	104.1 ± 80.9	153.6 ± 49.7	**0.0382**	132.9 ± 85.2	129.6 ± 68.8	0.7685	0.5127
**β-carotene (ug)**	1949.1 ± 947.6	2383.9 ± 406.1	**0.0005**	2127.9 ± 1056.1	2374.2 ± 385.9	0.2585	2226.2 ± 1097.5	2722.1 ± 355.8	**0.0937**	1552.6 ± 539.7	2114.4 ± 232.9	**0.0003**	0.0913
**Vitamin D (ug)**	4.5 ± 3.5	3.0 ± 2.2	**<0.0001**	3.8 ± 2.2	1.9 ± 1.4	**0.0002**	4.4 ± 3.3	3.4 ± 2.8	**0.1703**	5.2 ± 4.5	3.7 ± 2.0	**0.0495**	0.5204
**Vitamin E (ug)**	7.0 ± 3.9	11.1 ± 2.2	**<0.0001**	7.4 ± 3.0	11.6 ± 2.0	**<0.0001**	8.4 ± 5.4	11.8 ± 2.2	**0.0098**	5.6 ± 2.6	9.9 ± 1.9	**<0.0001**	0.1091
**Vitamin K (ug)**	40.1 ± 36.7	149.0 ± 39.2	**<0.0001**	42.5 ± 20.6	136.7 ± 9.8	**<0.0001**	49.3 ± 56.8	204.6 ± 15.0	**<0.0001**	30.3 ± 25.9	114.8 ± 7.3	**<0.0001**	0.3470
**Vitamin B1 (mg)**	1.2 ± 0.3	1.6 ± 0.3	**<0.0001**	1.1 ± 0.4	1.3 ± 0.2	**0.0142**	1.1 ± 0.4	1.8 ± 0.2	**<0.0001**	1.2 ± 0.3	1.6 ± 0.3	**0.0035**	0.5507
**Vitamin B2 (mg)**	1.2 ± 0.3	1.1 ± 0.2	**0.0251**	1.3 ± 0.4	1.2 ± 0.3	0.2853	1.2 ± 0.3	1.2 ± 0.2	**0.9760**	1.2 ± 0.2	1.1 ± 0.2	**0.0049**	0.7565
**Niacin (mg)**	11.4 ± 3.7	11.9 ± 1.9	0.4263	12.7 ± 4.6	10.6 ± 1.9	0.0703	10.5 ± 3.8	13.3 ±1.8	**0.0040**	11.1 ± 2.5	12.0 ± 0.9	0.1265	0.2490
**PA (mg)**	1.4 ± 0.8	2.4 ± 0.6	**<0.0001**	1.3 ± 0.4	2.2 ± 0.6	**<0.0001**	1.4 ± 1.0	2.4 ± 0.6	**<0.0001**	1.7 ± 1.0	2.6 ± 0.4	**<0.0001**	0.3639
**Vitamin B6 (mg)**	0.2 ± 0.1	0.4 ± 0.1	**<0.0001**	0.2 ± 0.1	0.4 ± 0.1	**0.0007**	0.2 ± 0.1	0.4 ± 0.1	**<0.0001**	0.2 ± 0.1	0.4 ± 0.1	**<0.0001**	0.4451
**Biotin (ug)**	8.4 ± 4.7	17.2 ± 3.4	**<0.0001**	7.8 ± 4.0	17.0 ± 3.3	**<0.0001**	8.3 ± 5.3	17.0 ± 3.5	**<0.0001**	9.1 ± 5.0	17.7 ± 3.5	**<0.0001**	0.7566
**Folate (ug)**	134.0 ± 58.0	220.4 ± 45.1	**<0.0001**	138.7 ± 59.0	206.2 ± 46.8	**<0.0001**	139.6 ± 58.7	243.2 ± 51.2	**<0.0001**	125.1 ± 59.0	215.2 ± 31.1	**<0.0001**	0.7383
**Vitamin B12 (ug)**	3.1 ± 2.7	2.3 ± 0.7	**0.0440**	3.6 ± 3.2	2.3 ± 0.5	0.1274	1.9 ± 1.5	2.3 ± 0.8	**0.2387**	3.6 ± 2.7	2.2 ± 0.8	**0.0484**	0.1366
**Vitamin C (mg)**	41.9 ± 61.0	66.3 ± 66.6	**<0.0001**	42.9 ± 36.9	60.3 ± 25.1	0.1209	66.5 ± 101.1	93.1 ± 117.1	**0.0337**	20.8 ± 9.9	49.8 ± 8.1	**<0.0001**	0.1145
**TS (g)**	35.9 ± 19.0	32.9 ± 12.4	0.1988	35.1 ± 20.4	29.6 ± 13.3	0.0981	39.1 ± 23.4	37.2 ± 10.8	**0.6966**	33.9 ± 13.8	32.5 ± 12.5	0.7273	0.7500
**TAA (mg)**	23.1 ± 10.1	22.7 ± 44.7	0.7156	24.4 ± 97.6	21.9 ± 39.7	0.2802	19.5 ± 11.5	20.0 ± 46.0	**0.8433**	24.9 ± 89.3	25.6 ± 30.8	0.6908	0.2815
**EAA (mg)**	10.9 ± 48.6	10.5 ± 21.3	0.5529	11.6 ± 45.6	10.0 ± 18.6	0.1642	89.9 ± 54.8	92.9 ± 21.6	**0.8032**	11.8 ± 44.1	12.0 ± 44.1	0.8265	0.2241
**NEAA (mg)**	12.3 ± 5.1	12.2 ± 23.4	0.8827	13.1 ± 49.1	11.8 ± 21.1	0.2632	10.4 ± 59.3	10.7 ± 24.4	**0.7738**	13.0 ± 45.3	13.6 ± 16.1	0.4956	0.2692
**TFA (g)**	26.6 ± 11.0	30.3 ± 5.4	**0.0411**	28.6 ± 10.6	27.9 ± 5.2	0.7992	21.8 ± 10.2	35.3 ± 4.8	**0.0002**	28.7 ± 11.4	28.5 ± 2.9	0.9278	0.1521
**EFA (g)**	6.1 ± 2.8	8.2 ± 1.2	**<0.0001**	6.3 ± 2.3	8.6 ± 1.3	**0.0001**	6.6 ± 3.9	8.6 ± 1.4	**0.0451**	5.4 ± 2.1	7.6 ± 0.6	**0.0004**	0.4742
**SFA (g)**	19.4 ± 6.1	11.0 ± 3.9	**<0.0001**	19.8 ± 6.7	8.1 ± 3.0	**<0.0001**	17.0 ± 6.0	15.4 ± 2.8	**0.3123**	20.9 ± 5.4	10.2 ± 1.6	**<0.0001**	0.1937
**MUFA (g)**	12.1 ± 4.9	16.8 ± 3.2	**<0.0001**	12.9 ± 5.4	14.4 ± 2.3	0.3031	10.2 ± 4.3	20.4 ± 2.3	**<0.0001**	13.0 ± 4.7	16.2 ± 1.7	**0.0125**	0.2066
**PUFA (g)**	6.5 ± 2.8	8.3 ± 1.2	**<0.0001**	6.9 ± 2.2	8.7 ± 1.3	**0.0008**	6.8 ± 4.0	8.6 ± 1.4	**0.0639**	6.0 ± 2.0	7.7 ± 0.6	**0.0031**	0.6106
**Water (g)**	782.8 ± 237.0	647.3 ± 140.4	**<0.0001**	802.6 ± 285.7	710.3 ± 168.7	0.0684	808.5 ± 262.9	630.3 ± 122.2	**0.0114**	742.9 ± 161.6	601.9 ± 107.0	**0.0016**	0.6934
**Ash (g)**	13.3 ± 3.7	11.0 ± 1.9	**<0.0001**	14.2 ± 4.2	11.6 ± 2.1	**0.0057**	13.3 ± 4.1	11.4 ± 1.9	**0.0671**	12.6 ± 2.8	10.1 ± 1.5	**0.0004**	0.4761
**Fiber (g)**	16.8 ± 6.1	21.1 ± 7.1	**0.0001**	17.9 ± 7.3	28.9 ± 6.2	**<0.0001**	16.9 ± 6.1	18.8 ± 3.0	**0.2755**	15.6 ± 4.8	15.7 ± 2.1	0.8935	0.5528
**Cholesterol (mg)**	292.2 ± 96.9	207.3 ± 70.3	**<0.0001**	289.2 ± 64.1	156.6 ± 47.3	**<0.0001**	247.5 ± 100.9	212.2 ± 76.8	**0.1512**	331.9 ± 106.9	250.9 ± 52.0	**0.0003**	0.0503
**Chlorine (mg)**	65.9 ± 69.4	138.2 ± 47.0	**<0.0001**	51.5 ± 37.8	100.9 ± 40.2	**<0.0001**	79.4 ± 99.7	154.3 ± 33.8	**0.0235**	68.3 ± 63.6	160.0 ± 42.3	**<0.0001**	0.5474
**Magnesium (mg)**	121.4 ± 48.4	146.0 ± 22.4	**<0.0001**	138.5 ± 55.2	141.8 ± 27.7	0.7311	120.7 ± 54.6	156.5 ± 20.2	**0.0098**	105.8 ± 29.9	141.3 ± 15.9	**<0.0001**	0.1525
**Fluorine (ug)**	2.9 ± 2.9	7.8 ± 2.5	**<0.0001**	2.7 ± 1.9	4.9 ± 0.7	**0.0004**	3.0 ± 3.1	8.3 ± 1.7	**<0.0001**	2.9 ± 3.5	10.2 ± 0.9	**<0.0001**	0.9629
**Iron (mg)**	20.3 ± 6.4	10.8 ± 3.2	**<0.0001**	20.4 ± 6.0	11.4 ± 3.8	**<0.0001**	20.0 ± 7.2	12.0 ± 2.9	**0.0023**	20.3 ± 6.6	9.2 ± 2.1	**<0.0001**	0.9815
**Zinc (mg)**	6.4 ± 2.4	6.0 ± 1.0	0.2046	6.8 ± 2.9	5.8 ± 1.0	0.1123	5.5 ± 2.4	6.3 ± 1.0	**0.1620**	6.7 ± 1.6	5.9 ± 0.9	0.0822	0.2605

**Copper (mg)**	0.4 ± 0.2	0.7 ± 0.1	**<0.0001**	0.5 ± 0.2	0.7 ± 0.1	**0.0007**	0.4 ± 0.2	0.7 ± 0.1	**<0.0001**	0.4 ± 0.1	0.7 ± 0.1	**<0.0001**	0.1803
**Manganese (mg)**	1.3 ± 0.7	1.9 ± 0.6	**<0.0001**	1.7 ± 0.8	2.4 ± 0.7	**<0.0001**	1.3 ± 0.5	1.8 ± 0.5	**0.0063**	1.1 ± 0.4	1.6 ± 0.2	**<0.0001**	**0.0303**
**Selenium (ug)**	49.6 ± 17.5	45.8 ± 9.7	0.1009	53.9 ± 19.2	44.7 ± 8.8	0.0651	41.2 ± 12.8	43.9 ± 11.9	**0.4014**	52.4 ± 17.7	48.4 ± 8.4	0.2687	0.0989
**Molyb-denum (ug)**	32.5 ± 21.8	38.0 ± 8.1	**0.0260**	46.8 ± 26.4	44.0 ± 9.1	0.5897	32.6 ± 17.2	36.9 ± 6.0	**0.3032**	19.0 ± 8.3	33.1 ± 4.5	**<0.0001**	**0.0005**

All values are expressed as mean ± SD. The *p*-values comparing specific timeline values at pre- and post-diet intervention in the total subjects, Group B, Group F, and Group P were measured by paired *t*-test. The *p*-values measured by ANOVA showed a significant difference in the pre-value among the three groups. Pre, before diet intervention; Post, after diet intervention. PA; pantothenic acid, TS; total sugars, TAA; total amino acids, EAA; essential amino acids, NEAA; non-essential amino acids, TFA; total fatty acid, EFA; essential fatty acid, SFA; saturated fatty acid, MUFA; mono-unsaturated fatty acid, PUFA; poly-unsaturated fatty acid.
